# Three-Dimensional Volumetric Measurement of Endolymphatic Hydrops in Meniere's Disease

**DOI:** 10.3389/fneur.2021.710422

**Published:** 2021-09-13

**Authors:** Tae-Soo Noh, Moo Kyun Park, Jun Ho Lee, Seung Ha Oh, Ji-Hoon Kim, In Chan Song, Myung-Whan Suh

**Affiliations:** ^1^Department of Otorhinolaryngology-Head and Neck Surgery, Seoul National University College of Medicine, Seoul National University Hospital, Seoul, South Korea; ^2^Department of Radiology, Seoul National University College of Medicine, Seoul National University Hospital, Seoul, South Korea

**Keywords:** endolymphatic hydrops, volumetric, planimetric, magnetic resonance imaging - MRI, menieres disease

## Abstract

**Objective:** We used volumetric three-dimensional (3D) analysis to quantitatively evaluate the extent of endolymphatic hydrops (EH) in the entire inner ear. We tested for correlations between the planimetric and volumetric measurements, to identify their advantages and disadvantages.

**Methods:** HYDROPS2-Mi2 EH images were acquired for 32 ears (16 patients): 16 ipsilateral ears of MD patients (MD-ears) and 16 contralateral ears. Three-T MR unit with a 32-channel phased-array coil/the contrast agent to fill the perilymphatic space and the HYDROPS2-Mi2 sequence. We calculated the EH% [(endolymph)/(endolymph+perilymph)] ratio and analyzed the entire inner ear in terms of the volumetric EH% value, but only single cochlear and vestibular slices were subjected to planimetric EH% evaluation. The EH% values were compared between MD ears and non-MD ears, to evaluate the diagnostic accuracy of the two methods.

**Results:** The volumetric EH% was significantly higher for MD vestibules (50.76 ± 13.78%) than non-MD vestibules (39.50 ± 8.99%). The planimetric EH% was also significantly higher for MD vestibules (61.98 ± 20.65%) than non-MD vestibules (37.22 ± 12.95%). The vestibular and cochlear volumetric EH% values correlated significantly with the planimetric EH% values of the MD ear.

**Conclusion:** Volumetric and planimetric EH measurements facilitate diagnosis of MD ears compared to non-MD ears. Both methods seem to be reliable and consistent; the measurements were significantly correlated in this study. However, the planimetric EH% overestimates the extent of vestibular hydrops by 26.26%. Also, planimetric data may not correlate with volumetric data for non-MD cochleae with normal EH% values.

## Introduction

Meniere's disease (MD) is characterized by repeated spells of vertigo accompanied by low-frequency hearing loss, hearing fluctuation, ear fullness, and tinnitus (1). In 2015, the Classification Committee of the Barany Society established guidelines for the diagnosis of MD. An MD patient should exhibit (A) two or more spontaneous episodes of vertigo (lasting 20 min to 12 h); (B) audiometrically documented, low-to medium frequency, sensorineural hearing loss in one ear; and (C) fluctuating aural symptoms (hearing, tinnitus, or fullness) in the affected ear (2). This standardized definition served as an important milestone for clinicians and researchers. However, all of the aforementioned criteria are subjective, or based on subjective hearing tests. As no criterion is objective, the diagnosis may sometimes be controversial or unclear. Classically, endolymphatic hydrops (EH) has been regarded as objective histopathological evidence of MD (3). However, histopathology can be performed only postmortem: it is not possible to evaluate a patient who is currently suffering from recurrent vertigo attacks. The time gap between the development of active MD and postmortem evaluation limits our understanding of how the disorder progresses. An objective diagnostic parameter would be very useful, especially when considering (invasive) intratympanic gentamicin injection, labyrinthectomy, or vestibular neurectomy.

Recently, magnetic resonance imaging (MRI) of EH has become possible (4, 5). The Nagoya group, among others, separated the perilymphatic and endolymphatic spaces in MR images. At least three different MRI techniques have been reported: (1) subtraction of two sequences with different inversion times; (2) turbo spin-echo inversion recovery via real reconstruction; and, (3) three-dimensional (3D)-fluid-attenuated inversion recovery (FLAIR) (6). All three techniques objectively imaged EH in MD patients (6–9).

EH imaging studies are promising but it is remains unclear how to objectively grade the extent of hydrops. Several grading systems have been developed to objectively classify the extent of EH; these vary by the imaging techniques used and the goals of the analysis. Most systems evaluate the relative size (planimetric ratio) of the endolymphatic area (mm^2^) in one or two slices of two-dimensional (2D) MR images (10–13). Slices including the mid-modiolar cochlear section and lower axial vestibule are typically analyzed. However, these approaches evaluate only a small proportion of the inner ear. As the inner ear has a complex 3D shape, and as some endolymphatic organs are not aligned along the axial plane, it may not be optimal to evaluate only one or two (supposedly representative) axial MRI slices. Some pioneering studies (14–16) sought to evaluate the relative 3D size (i.e., the volumetric ratio) of the endolymphatic volume (in μL) of the entire inner ear. However, these studies did not quantitatively compare the volumetric and planimetric EH ratios; a semi-quantitative approach was taken (16) but other studies (14, 15) lacked planimetric controls. Here, we explored the characteristics and advantages/disadvantages of volumetric EH measurements by directly and quantitatively comparing the volumetric and planimetric data.

## Methods

### Patients

Thirty-two ears were imaged in patients clinically diagnosed with definite (*n* = 11) or probable (*n* = 5) MD according to the 2015 criteria of the Classification Committee of the Barany Society (2). Patients with conditions that might affect MRI or hearing were excluded, as were those with a history of seizures, organic brain damage, or implantation of cardiac pacemakers, cochlear implants, or intraocular ferromagnetic materials. EH imaging was performed when MD was inactive, i.e., when no severe attack of dizziness had occurred within the prior month and hearing had been stable for at least 2 months. The gender ratio (M:F = 7:9), average age (47.3 ± 8.1 years) and duration of illness (53.6 ± 63.6 months) were similar to those of previous reports (17, 18). More detailed demographic data are listed in Tables 1, 2.

**Table 1:** Patient demographics.

	**Definite and probable Meniere's disease** **(***n*** = 16)**	**Definite Meniere's disease** **(***n*** = 11)**
Gender (M:F)	7:9	5:6
Age (years)	47.3 ± 8.1	42.6 ± 6.2
MD subtype (definite:probable)	11:5	11:0
MD side (R:L)	4:12	4:7
Dizziness duration (min)	41.9 ± 59.1	61.2 ± 64.7
Duration of illness (months)	53.6 ± 63.6	56.5 ± 76.9
Tinnitus (Y:N)	11:5	7:4
Ear fullness (Y:N)	10:6	7:4
Hearing fluctuation (Y:N)	8:8	4:7

*MD, Meniere's disease; Data are expressed as means ± standard deviations (SDs)*.

**Table 2:** Pure-tone audiometry thresholds.

	**Definite and probable Meniere's disease** **(***n*** = 16)**			**Definite Meniere's disease** **(***n*** = 11)**		
**Frequency**	**MD side**	**Non-MD side**	***P*** **-value**	**MD side**	**Non-MD side**	***P*** **-value**
250 Hz	52.4 ± 25.1	11.1 ± 7.2	<0.001	60.9 ± 20.7	10.5 ± 7.9	<0.001
500 Hz	54.5 ± 26.6	13.9 ± 7.9	<0.001	65.5 ± 22.3	14.1 ± 8.3	<0.001
1,000 Hz	53.2 ± 25.9	11.8 ± 8.4	<0.001	65.0 ± 20.6	11.4 ± 9.8	<0.001
2,000 Hz	49.2 ± 24.1	13.4 ± 9.9	<0.001	57.3 ± 21.3	13.6 ± 10.3	<0.001
4,000 Hz	54.5 ± 23.3	21.3 ± 14.9	<0.001	57.3 ± 19.4	23.2 ± 14.2	<0.001
8,000 Hz	64.5 ± 15.4	26.6 ± 23.1	<0.001	65.5 ± 12.1	28.6 ± 24.8	0.002

*The data are means ± standard deviations*.

### MRI

Four hours after injection of contrast agent (Magnevist; Bayer Ltd., Leverkusen, Germany), MRI scans were performed using a 3-T MR unit (3-T Magnetom Tim Trio; Siemens Medical Solutions, Erlangen, Germany) with a 32-channel phased-array coil (4). All MRI protocols were those of the Nagoya group (19). Patients underwent heavily T2-weighted (hT2w) MR cisternography (MRC) to locate the inner ear, in addition to hT2w 3D-FLAIR with an inversion time of 2,250 ms (yielding a perilymph-positive image, PPI). For the variable flip angle 3D-turbo spin-echo technique, termed SPACE (sampling perfection with application-optimized contrasts by using different flip angle evolutions), the parameters were as follows: repetition time (TR) 4,400 ms; echo time (TE) 546 ms; initial refocusing at 180° with the flip angle then rapidly decreased to a constant 120° for turbo spin-echo refocusing of the echo train; echo train length, 203 (with a restorative magnetization pulse, i.e., a fast recovery pulse); matrix size, 319 × 384; 104 1.0-mm-thick axial slices covering the labyrinth; field of view (FOV), 15 × 18 cm; use of the generalized auto-calibrating, partially parallel acquisition (GRAPPA) parallel imaging technique; acceleration factor, 2; number of excitations (NEX), four; and scan time, 6 min 30 s. The PPI scan parameters of hT2W-3D-FLAIR were similar to those of MRC, except for application of an inversion pulse with an inversion time of 2,250 ms; the TR was 9,000 ms, the NEX was 4, and the scan time was 15 min 32 s. The PPI did not feature a restorative pulse. The FOVs, matrix sizes, and slice thicknesses of MRC and PPI were identical, to facilitate comparisons. HYDROPS2 images were generated by subtracting each MRC image (multiplied by 0.05) from the PPI. Negative HYDROPS2 images were acceptable. Acquisition of the source HYDROPS2 images took 18 min. Each HYDROPS2-Mi2 image was obtained by multiplying the MRC and HYDROPS2 images (19).

### Endolymphatic Hydrops Analysis

We analyzed the HYDROPS2-Mi2 EH images of 32 ears (16 ipsi-lesional MD ears and 16 contralateral non-MD ears). The perilymph and endolymph were clearly demarcated in all 32 ears. We used a threshold technique based on the signal intensity of HYDROPS2-Mi2 images to quantitatively analyze the endo- and peri-lymphatic space volumes, which were segmented as negative (< −1) and positive (>5) threshold signal intensities, respectively, on manually drawn regions of interest (ROIs) of the cochlea and vestibule evident on MR cisternographs. Although the signal intensities of bony structures in ROIs are set to zero in HYDROPS2-Mi2 images, any remaining bony structures within cochleae and vestibules may be of non-zero intensity were removed. We used cutoff values of−1 and 5 to minimize volume overestimations near the boundaries of the cochlear and vestibular systems.

**Figure 1:**
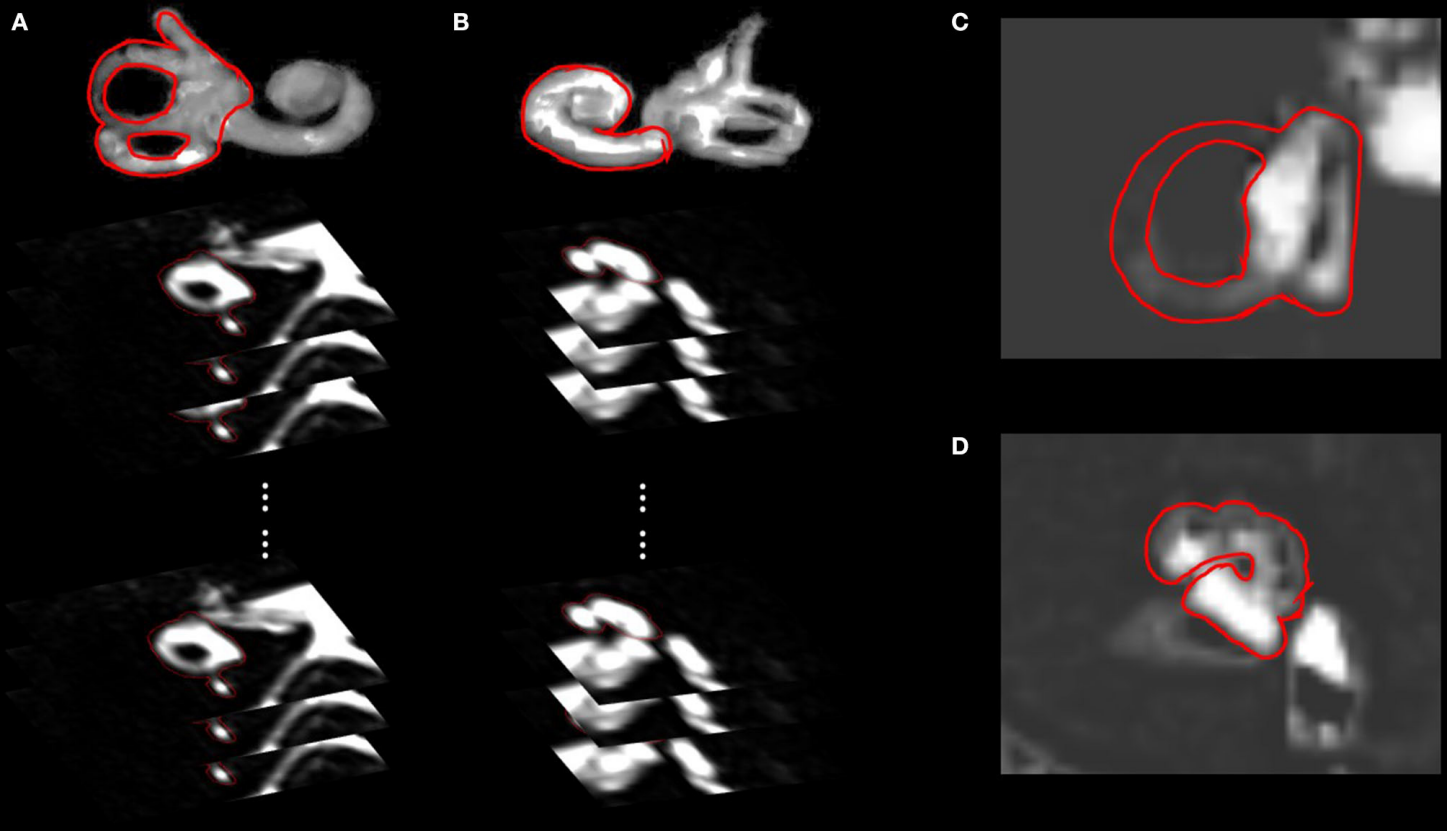
Regions of interests (ROIs) of the volumetric and planimetric analyses. Volumetric analyses considered the whole inner ear (10–15 slices). By analyzing all images that included the vestibule **(A)** or cochlea **(B)**, we obtained the volumes (in μL) of the endolymphatic and perilymphatic spaces. Planimetric analysis measured the areas (in mm^2^) of vestibules **(C)** and cochleae **(D)** in a single representative slice, as suggested by Naganawa et al. The lowest slice, in which over 240° of the lateral semicircular canal ring was visible, was taken to represent the vestibule **(C)**, and the slice with the largest cochlear modiolus represented the cochlea **(D)**.

For volumetric analysis, all MR images (10–15 slices) covering the vestibule (Figure 1A) and cochlea (Figure 1B) were 3D-stacked. The absolute volumes (in μL) of the endolymph and perilymph were compared between MD and non-MD ears. The quantitative volumetric EH% (endolymph volume (μL))/(endolymph+perilymph volume (μL)) was calculated automatically by the software. For conventional planimetric analysis, two representative cross-sectional MR images were analyzed by drawing cochlear and vestibular ROIs (Figures 1C,D) using the method of (4). For the vestibular ROI, the lowest slice wherein over 240° of the lateral semicircular canal ring was apparent was selected (Figure 1C). For the cochlear ROI, the slice exhibiting the largest cochlear modiolus was selected (Figure 1D). The absolute areas (in mm^2^) of the endolymph and perilymph were compared between MD and non-MD ears. The quantitative planimetric EH% [endolymph area (mm^2^)/endolymph+perilymph area (mm^2^)] was calculated. MD and non-MD ears were compared to determine if the volumetric and planimetric analyses identified the pathological side. Also, the volumetric and planimetric EH% values were compared within each subject.

### Statistical Analysis

Continuous variables are expressed as means ± standard deviations (SDs). All statistical analyses were performed using SPSS software (ver. 25.0; SPSS Inc., Chicago, IL, USA). The Wilcoxon test was used to compare the volumetric and planimetric data. Also, Mann–Whitney U test was used to compare the EH% between definite and probable MD patients. Correlations were derived using the Spearman method. A *p*-value < 0.05 was considered statistically significant.

## Results

### Vestibular Endolymphatic Hydrops

**Figure 2:**
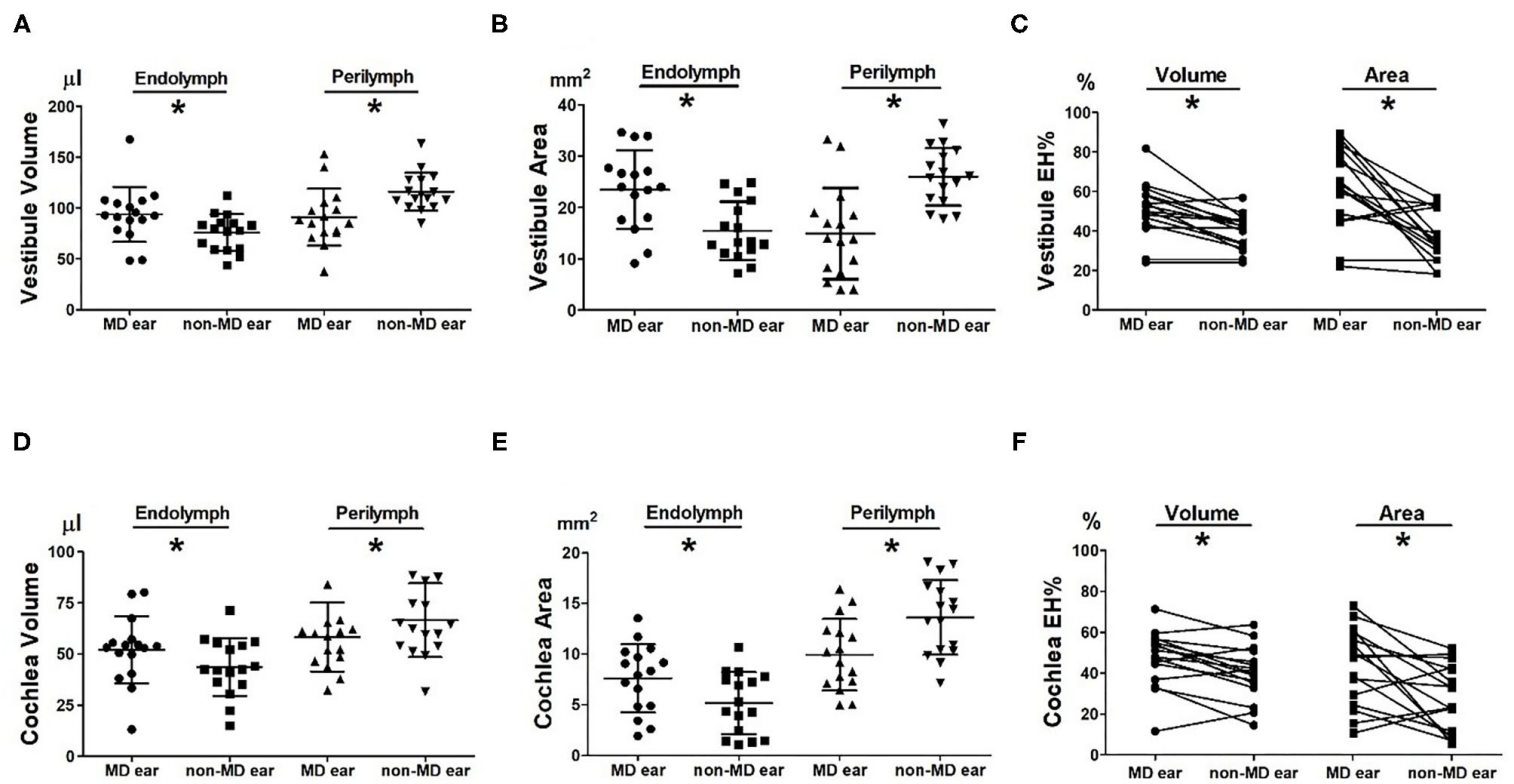
Comparison of volumetric (μL) and planimetric (mm^2^) measurements between MD and non-MD ears. The endolymph volume and area were significantly larger in MD than non-MD ears **(A,B,D,E)**. In contrast, the perilymph volume and area were significantly lower in MD than non-MD ears. Thus, the volumetric and planimetric EH% values (the percentages of endolymphatic hydrops) were significantly greater in MD ears than non-MD ears **(C,F)**. The EH% difference between MD and non-MD ears was more pronounced on planimetric than volumetric measurement.

looseness1Figures 2A,C compare the volumetric data of the MD and non-MD vestibules. The vestibular endolymph volume was significantly greater in MD ears (93.56 ± 27.15 μL) than in non-MD ears (75.73 ± 18.00 μL; *p* = 0.004, Figure 2A). In contrast, the volume of the vestibular perilymph was significantly lower in MD ears (91.01 ± 27.90 μL) compared to non-MD ears (115.86 ± 18.67 μL; *p* = 0.001, Figure 2A). The volumetric EH% was significantly larger in MD vestibules (50.76 ± 13.78%) than non-MD vestibules (39.50 ± 8.99%, *p* = 0.001, Figure 2C). The volumetric EH% objectively distinguished the pathological side with an accuracy of 93.75% (15/16); with the exception of one subject, the EH% was always larger in the MD vestibule (mean difference, 11.26 ± 10.29%).

looseness1Figures 2B,C compare the planimetric data of the MD and non-MD vestibules. The area of vestibular endolymph was significantly greater in MD ears (23.47 ± 7.63 mm^2^) than in non-MD ears (15.43 ± 5.67 mm^2^; *p* = 0.008, Figure 2B). In contrast, the area of vestibular perilymph was significantly smaller in MD ears (14.91 ± 8.86 mm^2^) than non-MD ears (25.96 ± 5.60 mm^2^; *p* = 0.002, Figure 2B). The planimetric EH% was significantly larger in MD vestibules (61.98 ± 20.65%) than non-MD vestibules (37.22 ± 12.95%; *p* = 0.003, Figure 1C). The planimetric EH% objectively distinguished the pathological side with an accuracy of 81.25% (13/16); with the exception of three subjects, the EH% was larger in the MD vestibule (mean difference, 30.70 ± 36.93%).

### Cochlear Endolymphatic Hydrops

Figures 2D,F compare the volumetric data of the MD and non-MD cochleae. The volume of cochlear endolymph in MD ears (52.11 ± 16.39 μL) was significantly greater than that in non-MD ears (43.62 ± 14.16 μL; *p* = 0.005, Figure 2D). In contrast, the volume of cochlear perilymph in MD ears (58.25 ± 16.82 μL) was significantly lower than that in non-MD ears (66.47 ± 18.01 μL; *p* = 0.023, Figure 2D). The volumetric EH% of the MD cochlea (47.29 ± 13.84%) was significantly larger than that of the non-MD cochlea (39.98 ± 13.21%; *p* = 0.008, Figure 1F). The volumetric EH% identified the pathological ear with an accuracy of 75.00% (12/16) (mean difference, 7.31 ± 9.26%).

Figures 2E,F compare the planimetric data of the MD and non-MD cochleae. The area of cochlear endolymph was significantly greater in MD ears (7.62 ± 3.35 mm^2^) than in non-MD ears (5.17 ± 3.05 mm^2^; *p* = 0.044). In contrast, the area of cochlear perilymph was significantly smaller in MD ears (9.94 ± 3.52 mm^2^) than in non-MD ears (13.62 ± 3.69 mm^2^; *p* = 0.005). The planimetric EH% was significantly larger for MD cochleae (43.52 ± 18.96%) than for non-MD cochleae (27.80 ± 16.29%; *p* = 0.010, Figure 1F). The planimetric EH% identified pathological cochleae with an accuracy of 68.75% (11/16) (mean difference, 15.71 ± 20.53%).

### Correlations Between Volumetric and Planimetric Data

**Figure 3:**
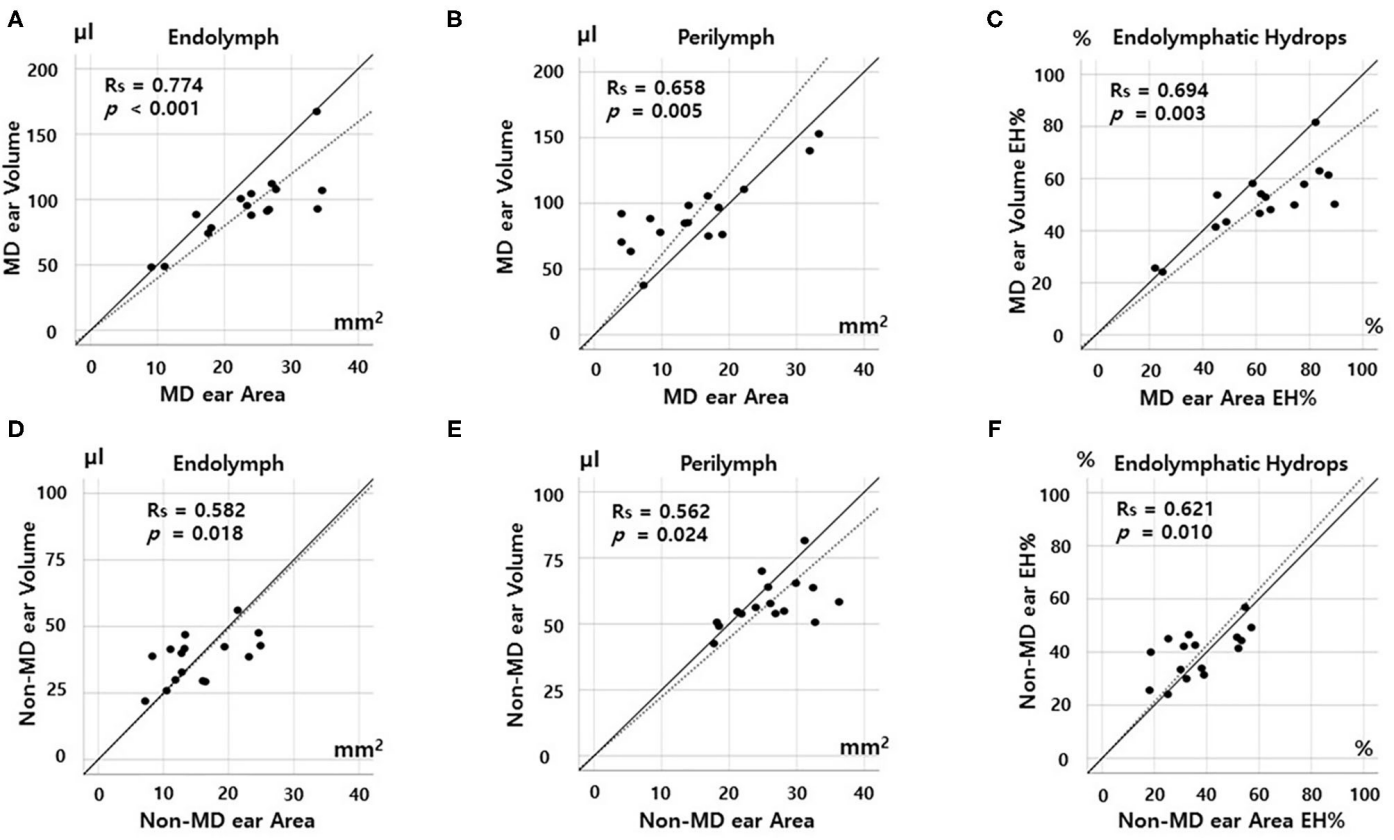
Correlation between volumetric (μL) and planimetric (mm^2^) measurements of the vestibule. In the Meniere's disease (MD) vestibule, the endolymph and perilymph volumes correlated significantly with the planimetric measurements **(A,B)**. The volumetric EH% value (the percentage of endolymphatic hydrops) correlated significantly with the planimetric EH% value **(C)**. The planimetric EH% values were greater (data points below the solid diagonal line) in most subjects **(C)**. Regression modeling revealed that the planimetric measurement overestimated the EH% by 26.26% (volumetric EH% = planimetric EH% ^*^0.792). Good correlations were also evident for the non-MD vestibule: The volumetric and planimetric endolymph, perilymph, and EH% values were significantly correlated **(D–F)**.

**Figure 4:**
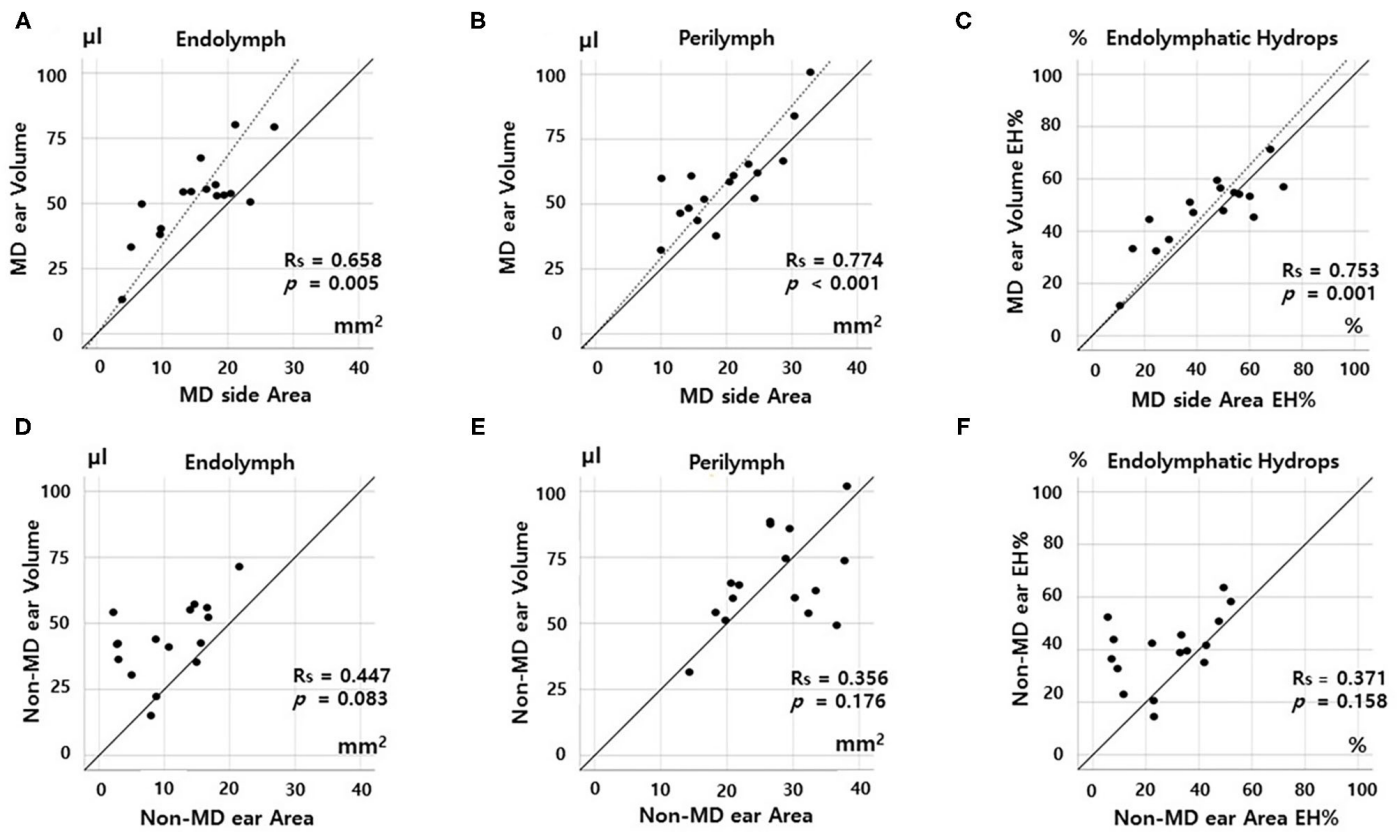
Correlations between volumetric (μL) and planimetric (mm^2^) measurements of the cochlea. In the MD cochlea, the endolymph and perilymph volumes correlated significantly with the planimetric measurements **(A,B)**. The volumetric EH% also correlated significantly with the planimetric EH% **(C)**. However, the correlation was weak for non-MD cochleae; there were no correlations among the endolymph volumes, perilymph volumes, or EH% values of volumetric and planimetric measurements **(D–F)**.

Figures 3, 4 show the correlations between the volumetric and planimetric data of the vestibule and cochlea, respectively. In MD vestibules, the volumes of endolymph (Rs = 0.774, *p* < 0.001) and perilymph (Rs = 0.658, *p* = 0.005) were significantly correlated with the planimetric measurements (Figures 3A,B). The volumetric EH% was also significantly correlated with the planimetric EH% (Rs = 0.694, *p* = 0.003, Figure 3C). In the regression model, the planimetric measurements overestimated the EH% by 26.26% (volumetric EH% = planimetric EH% ^*^ 0.792). Good correlations were also evident for non-MD vestibules: the endolymph (*r* = 0.582, *p* = 0.018), perilymph (Rs = 0.562, *p* = 0.024), and EH% parameters (Rs = 0.621, *p* = 0.010) were significantly correlated between the volumetric and planimetric measurements (Figures 3D–F).

In the MD cochlea, the volumes of the endolymph (Rs = 0.668, *p* = 0.005) and perilymph (Rs = 0.774, *p* < 0.001) correlated significantly with the planimetric measurements (Figures 4A,B). The volumetric EH% also significantly correlated with the planimetric EH% (Rs = 0.753, *p* = 0.001, Figure 3C, volumetric EH% = planimetric EH% ^*^ 1.013). However, weak correlations were seen for non-MD cochleae (endolymph, Rs = 0.447, *p* = 0.083; perilymph, Rs = 0.356, *p* = 0.176; and EH%, Rs = 0.371, *p* = 0.158). That is, the volumetric and planimetric data were not correlated in the non-MD cochleae (Figures 4D–F).

### Comparison Between Definite MD and Probable MD Patients

**Figure 5:**
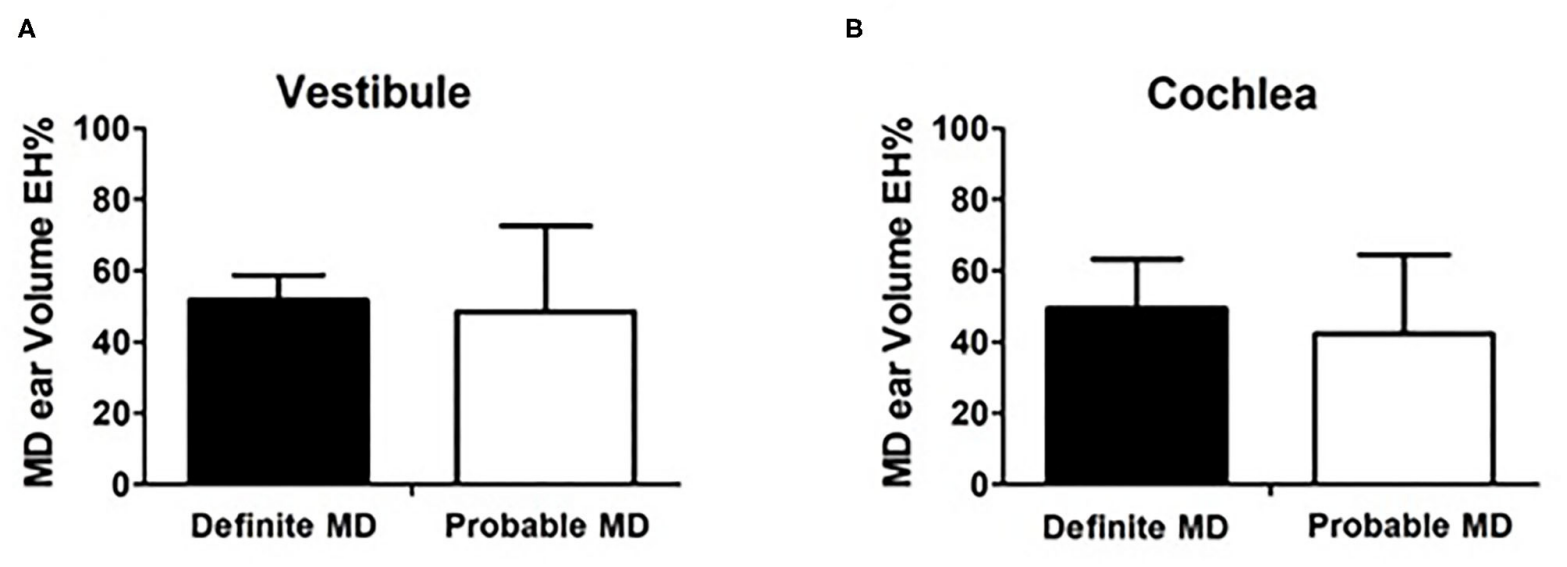
Comparison between definite MD and probable MD patients. **(A,B)** compare the endolymphatic hydrops percentage (EH%) between the two groups. The volumetric vestibular EH% was similar between definite MD (51.79 ± 6.97 μL) and in probable MD (48.50 ± 24.11 μL; *p* = 0.913, **A**). The volumetric cochlear EH% was also similar between the definite MD (49.54 ± 13.71 μL) and probable MD (42.36 ± 22.07 μL; *p* = 0.377, **B**) group.

Figures 5A,B compare the endolymphatic hydrops percentage (EH%) between the two groups. The volumetric vestibular EH% was similar between definite MD (51.79 ± 6.97 μL) and in probable MD (48.50 ± 24.11 μL; *p* = 0.913, Figure 5A). The volumetric cochlear EH% was also similar between the definite MD (49.54 ± 13.71 μL) and probable MD (42.36 ± 22.07 μL; *p* = 0.377, Figure 5B) group.

## Discussion

### Quantitatively Compare the Volumetric and Planimetric EH Ratios

Single vestibular and cochlear slices adequately represented the 3D EH status of the entire inner ear. The volumetric data correlated significantly with the conventional planimetric data (Figures 3C, 4C) of MD ears, which exhibited significantly higher EH% values than did non-MD ears (Figures 2C,F). EH imaging objectively distinguished the pathological side both volumetrically (accuracy, 75–94%) and planimetrically (accuracy, 68–81%). Given the need for complicated post-processing of volumetric data, this is encouraging. Although underestimation of EH volume may be of scientific concern, conventional planimetric measurements suffice in everyday practice. This is the first report to describe significant correlations between quantitative volumetric and planimetric analyses.

Certain differences between the two methods require consideration. First, the planimetric EH% overestimated the extent of vestibular hydrops by 26.26%. As shown in Figure 1C, planimetric measurements tend to inflate the EH% values of MD, but not non-MD, ears. Possible overestimation of hydrops volume on planimetric analysis was mentioned in a previous report (16). The single MRI slice that is planimetrically analyzed includes the anatomical location where hydrops is most pronounced. Thus, this single slice is exceptional. This may be an advantage of planimetric analysis; it becomes easier to distinguish MD and non-MD ears (Figures 1C,F). However, such increased sensitivity may decrease the specificity of MRI-based EH diagnosis. Also, it is important to not misinterpret the planimetric EH% value. For example, a planimetric EH of 89.40% (in subject 2 of Figure 2C) does not mean that 89.40% of the inner ear volume is filled with endolymph (the volumetric value was only 50.17%). However, the regression formula (volumetric EH% = planimetric EH% ^*^ 0.792) allows the volumetric EH% of the entire inner ear to be simply determined using a planimetric EH% value derived from one MRI slice.

Second, the vestibular correlations were better than the cochlear correlations. As shown in Figure 4F, the EH% values of the volumetric and planimetric analyses were not correlated in non-MD cochleae, probably because the cochlear duct is a spiraled narrow tube, whereas the vestibule has a simple 3D structure. Endolymph and perilymph imaging and demarcation are more challenging in the cochlea. Also, the cochlea seems to be less affected by hydrops because the tight surrounding structures (including the bony spiral lamina) restrict cochlear expansion. Especially, we found no correlations between parameters in non-MD (normal) cochlea (Figure 3F) because the data is not spread out. Compared to that of the vestibule, EH imaging data of the cochlea may be difficult to clinically interpret. Similarly, other studies found that the cochlear EH was not well-correlated with clinical findings, such as the hearing threshold (20).

Many studies have sought to use EH imaging to diagnose MD (4, 10, 18, 21). Most studies reported more EH in affected ears, but the simplistic nature of the analyses created a great deal of controversy and some tension (6, 22). We found that the conventional planimetric method of the Nagoya group was reliable and consistent, being both simple and well-reflecting the volumetric EH of the entire inner ear. The EH% results did not differ by measurement type (volumetric or planimetric analysis).

### MD Diagnosis Issue Using EH Imaging

EH imaging aids objective MD diagnosis (4). However, there are certain underlying issues. First, the resolution of MRI is relatively low (voxel size, 0.47 × 0.47 × 1.00 mm^3^) (23) compared to the size of the inner ear (180–300 mm^3^) (14, 16, 24). Thus, it may be difficult to accurately calculate the hydrops volume, regardless of the post-processing or analysis methods used. The boundary between endolymph and perilymph may be blurred; the curvature of the cochlea or the canals may not be smooth; and the volume of the inner ear may vary depending on the imaging technique used. Second, EH may not be a pathognomonic sign of MD. Some authors have suggested that hydrops may be common to various inner ear disorders, including vestibular neuritis and vestibular migraine (20, 25). Hydropic ear disease (HED), which encompasses a wider spectrum of EH, including clinical variants and primary and secondary MD, may be a more appropriate diagnosis (26). Third, the long waiting and imaging times can be problematic. It takes 4 h for the contrast agent to fill the perilymphatic space and the HYDROPS2-Mi2 sequence requires at least 30 min of MRI (4).

### Limitation

Our work had certain limitations. First, the number of subjects was small; more subjects would strengthen our conclusions. Also, we lacked a control group (normal subjects with no inner ear symptoms). However, as our aim was to compare the volumetric and planimetric EH% values within subjects, these limitations do not undermine our conclusions. Not all subjects were diagnosed with definite MD using the criteria of the Barany Society. As in other EH imaging studies (25, 27, 28), a few subjects fulfilled the diagnostic criteria of probable MD.

## Conclusion

EH volumetric and planimetric measurements facilitate objective distinction of MD from non-MD ears. Both methods are reliable and consistent; the measurements correlate significantly. Although conventional planimetric analysis considers only one or two MRI slices, it nonetheless reflected the volumetric EH of the entire inner ear in this study. But it should be noted that the conventional planimetric measurement overestimated vestibular hydrops by 26.26%. Also, planimetric and volumetric cochlear data were not correlated in subjects with normal EH% values.

## Data Availability Statement

The original contributions presented in the study are included in the article/supplementary material, further inquiries can be directed to the corresponding author/s.

## Ethics Statement

The studies involving human participants were reviewed and approved by institutional review board at Seoul National University Hospital (1212-081-451 and 1806-047-950). The patients/participants provided their written informed consent to participate in this study. Written informed consent was obtained from the individual(s) for the publication of any potentially identifiable images or data included in this article.

## Author Contributions

MP, JL, and SO contributed to the design conception of the study, collected, and analyzed the data. T-SN, IS, and M-WS contributed to data analysis and the writing of the manuscript. J-HK and M-WS contributed to the design conception of the study and data analysis. All authors contributed to the article and approved the submitted version.

## Funding

This research was supported by the Korea Health Industry Development Institute (KHDI) of Korean Ministry of Health and Welfare (HI18C0626).

## Conflict of Interest

The authors declare that the research was conducted in the absence of any commercial or financial relationships that could be construed as a potential conflict of interest.

## Publisher's Note

All claims expressed in this article are solely those of the authors and do not necessarily represent those of their affiliated organizations, or those of the publisher, the editors and the reviewers. Any product that may be evaluated in this article, or claim that may be made by its manufacturer, is not guaranteed or endorsed by the publisher.
